# Pediatric Spinal Neuroschistosomiasis in Yemen: An Overlooked Dilemma in an Endemic Region

**DOI:** 10.7759/cureus.41758

**Published:** 2023-07-12

**Authors:** Nabil Aljuma'ai, Saif A Ghabisha, Faisal Ahmed, Taha Al-Mwald, Abdullah Mayas, Bushra Almaghribi, Hamzah Esmail, Mohamed Badheeb

**Affiliations:** 1 Department of Pediatrics, Ibb University, Ibb, YEM; 2 Department of Pediatrics, Pediatric Consultation Clinic, Ibb, YEM; 3 Department of General Surgery, Ibb University, Ibb, YEM; 4 Department of Urology, Ibb University, Ibb, YEM; 5 Department of Radiology, Ibb University, Ibb, YEM; 6 Radiology, Yemen Scan Center, Ibb, YEM; 7 Department of Internal Medicine, Hadhramaut University, Hadhramaut, YEM

**Keywords:** spinal cord, schistosoma, pediatrics, neuroschistosomiasis, magnetic resonance imaging, cerebrospinal fluid

## Abstract

Background: Neurological involvement in schistosomiasis presents a significant and serious complication. While the disease is generally considered treatable during the early stages, the rarity of this condition often leads to delays in diagnosis and treatment. This study aims to report the clinical characteristics of pediatric patients with spinal neuroschistosomiasis (NS) in an endemic area to the disease.

Methods: A retrospective cross-sectional review was conducted at Althora General Hospital in Ibb, Yemen, from January 2016 to January 2021. The study examined confirmed pediatric cases of spinal NS, analyzing their clinical characteristics, laboratory and radiological data, treatment approaches, and complications.

Results: The study identified 10 cases of spinal NS with a mean age of 10.1± 3.2 years. The majority (90%) were male and from rural areas, all with a history of freshwater exposure, a known risk factor for schistosomiasis. The average time from presentation to treatment was 33.4± 45.6 days (7-150 days). Common symptoms observed in all patients were bladder dysfunction and paresthesia (100%). Intestinal dysfunction was prevalent in 90% of cases, while 80% exhibited limb weakness or inability to walk. The diagnosis was confirmed through cerebrospinal fluid (CSF) serology in 80% of cases, and stool and urine exams yielded positive results in 90% and 30% of cases, respectively. Magnetic Resonance Imaging findings revealed medullary lesions in 50% of cases, cauda equina lesions in 20%, and multiple lesions in 30%. All patients received oral praziquantel and high-dose steroids for at least three days as part of their initial treatment. During the average follow-up period of 5.6±1.7 months, one patient experienced lower extremity paraplegia, while two cases (20%) showed partial improvement with residual deficits including urinary and fecal incontinence. Complete resolution of symptoms was achieved in seven cases (70%).

Conclusion: Schistosomiasis should be considered in pediatric patients with myeloradicular manifestations, especially in endemic areas. Early identification can be achieved through history, prompt imaging, and CSF serology. In the absence of immediate test results, expert-guided presumptive therapy should be considered to minimize neurological complications.

## Introduction

Schistosomiasis, a systemic disease known for its predominant involvement in the urological and gastrointestinal tracts, encompasses a wide range of manifestations [1]. Nonetheless, the dissemination of trematode eggs into the systemic circulation through the portal or splenic veins enables the involvement of distant organs such as the kidneys, heart, testicles, and the neurological system [1-3]. Alarming statistics reveal a persistently high burden of schistosomiasis, affecting more than 200 million individuals globally, with the highest prevalence documented in African and Asian regions. Of particular concern is the estimation that around one-third of school children in Yemen are afflicted by schistosomiasis [4].

Neuroschistosomiasis (NS) represents a severe and incapacitating form of schistosomiasis that primarily arises from infections caused by *S. mansoni*, *S. japonicum*, *S. mekongi*, and *S. haematobium*, prevalent in regions endemic to the disease [5]. The dissemination of schistosomes is predominantly observed in the cerebrum and, to a lesser extent, the spinal cord [5]. Nevertheless, spinal involvement tends to exhibit symptomatic manifestations in earlier stages, presenting with a diverse range of symptoms that can vary from nonspecific to severe and life-threatening, such as transverse myelitis accompanied by hemorrhagic and necrotic complications [5,6]. Encouragingly, the timely administration of praziquantel and high-dose steroids has demonstrated efficacy in mitigating the neurological sequelae associated with spinal NS. However, the optimal therapeutic approach for NS lacks consensus guidelines or clinical trials. Current strategies involve a combination of antiparasitic agents, corticosteroids, and, in select cases, surgical intervention. Overall, the management of NS requires an individualized approach based on the severity of symptoms and clinical presentation [5,6].

The available literature on spinal cord NS is limited, particularly in our country, and the number of pathologically confirmed cases remains primarily confined to isolated case reports or small case series [1,7]. Therefore, this study aims to provide a comprehensive report on the clinical pattern of pediatric patients diagnosed with spinal NS in an endemic area located in Ibb City, Yemen.

## Materials and methods

Study design

A retrospective cross-sectional review was conducted on confirmed pediatric cases of spinal NS referred to Althora General Hospital in Ibb, Yemen, covering the period from January 2016 to January 2021. Ethical approval for the study was obtained from the Ethics Research Committees of Ibb University (ID: IBBUNI.AC.YEM.2023.75, issued on 07/03/2023).

The diagnosis of spinal NS was established through a comprehensive assessment of clinical and radiological findings, as well as available parasitological results. Serum, CSF, urinary, and stool specimens were subjected to biochemical and cytomorphological examinations. Furthermore, patients were assessed for other potential causes of transverse myelitis, with various serological tests including complete blood cell count (CBC), glucose levels, cyanocobalamin, lupus anticoagulant, antinuclear antibodies, Venereal Disease Research Laboratory (VDRL) test. Additionally, hepatitis B, cytomegalovirus, herpes simplex virus, human T lymphotropic virus types 1 and 2, and HIV profiles were obtained. Furthermore, radiological evaluations involved brain and spinal magnetic resonance imaging (MRI). In addition, abdominal ultrasound (US) examinations were performed on all patients to evaluate the presence of periportal fibrosis and portal hypertension.

Data collection, treatment, and outcome

Patients' clinical and demographic data were obtained through the available medical records, in addition to their illness onset, progression, course while admitted, laboratory and radiological testing results, received therapy, follow-up, and outcomes.

CSF examination was considered significant with elevated WBC (>4 cells/mm^3^) and protein (> 40 mg/dl) measurements. In the current study, a therapeutic regimen consisting of praziquantel was implemented at four different time points over the course of one year. The prescribed dosage was 40 mg/kg/day, administered in three divided doses. This dosage was repeated for three consecutive days and followed by the same regimen every four months for a minimum of one year. Additionally, prednisone was administered at an initial dose of 40 mg/day, gradually tapered over a few weeks [8]. After treatments, physical therapy was recommended for all patients.

Patients' therapeutic responses and outcomes were assessed to determine their level of recovery. Complete recovery was defined as the absence of reported neurological symptoms and the absence of any neurological findings during a clinical examination. Partial recovery was assigned to patients who exhibited residual symptoms or clinical findings, and no extent of functional impairments impacting their daily activities was considered. No recovery was indicated when minimal or insignificant improvement was observed, and no significant restoration of neurological function was evident [9]. Additionally, the patient's outcome (fully improved vs. partially improved or no recovery) was compared based on the time elapsed from the initial presentation till the administration of definitive therapy.

Statistical analysis

Statistical analyses were performed using IBM SPSS version 22 software (IBM Corp., Armonk, New York). Descriptive statistics were used to summarize the data, with quantitative variables presented as means accompanied by standard deviations, while qualitative variables were reported as frequencies and percentages. The Fisher exact test was utilized to compare the variable of interest, and a significance level of p < 0.05 was deemed statistically significant.

## Results

A total of 10 cases of spinal NS were identified. The mean age at diagnosis was 10.1 ± 3.2 years, with a median age of 9 years, and the range was between 7 and 17 years. The majority of cases (90%) were male patients from rural areas. All patients had a history of freshwater exposure, indicating epidemiological risk factors for schistosomiasis. The mean time from presentation till definitive therapy was 33.4 ± 45.6 days, with a median time of 11.5 days (minimum: 7 days, maximum: 150 days). Various clinical symptoms were observed among the cases, including rapidly progressive weakness (present in 60% of cases), bladder dysfunction (100%), intestinal dysfunction (90%), lower limb muscle weakness (80%), fever (60%), inability to walk (80%), and abdominal pain (60%). Paresthesia was reported in all cases (100%), while deep tendon hyporeflexia and areflexia were noticed in 40% and 20% of patients, respectively. The abdominal US revealed a periportal hyperechogenic signal in two (20%) of the patients and hepatosplenomegaly in two (20%) but no indication of portal hypertension (Table 1).

**Table 1:** Clinical characteristics and physical examination of pediatric patients with spinal schistosomiasis.

Variables	N (%)
Age (year), Mean± SD	10.1±3.2 (range 7 – 17)
Gender	
Male	9 (90%)
Female	1 (10%)
Residency	
Rural	9 (90%)
Urban	1 (10%)
Duration of symptoms (days), Mean± SD	33.4±45.6 (Min: 7 - Max: 150 days)
Symptoms	
Bladder dysfunction	10 (100%)
Paresthesia	10 (100%)
Intestinal dysfunction	9 (90%)
Inability to walk	8 (80%)
Lower limb muscle weakness	8 (80%)
Rapidly progressive weakness	6 (60%)
Fever	6 (60%)
Abdominal pain	6 (60%)
Deep tendon Hyporeflexia	4 (40%)
Deep tendon Areflexia	2 (20%)
Hepatosplenomegaly	2 (20%)

**Figure 1:**
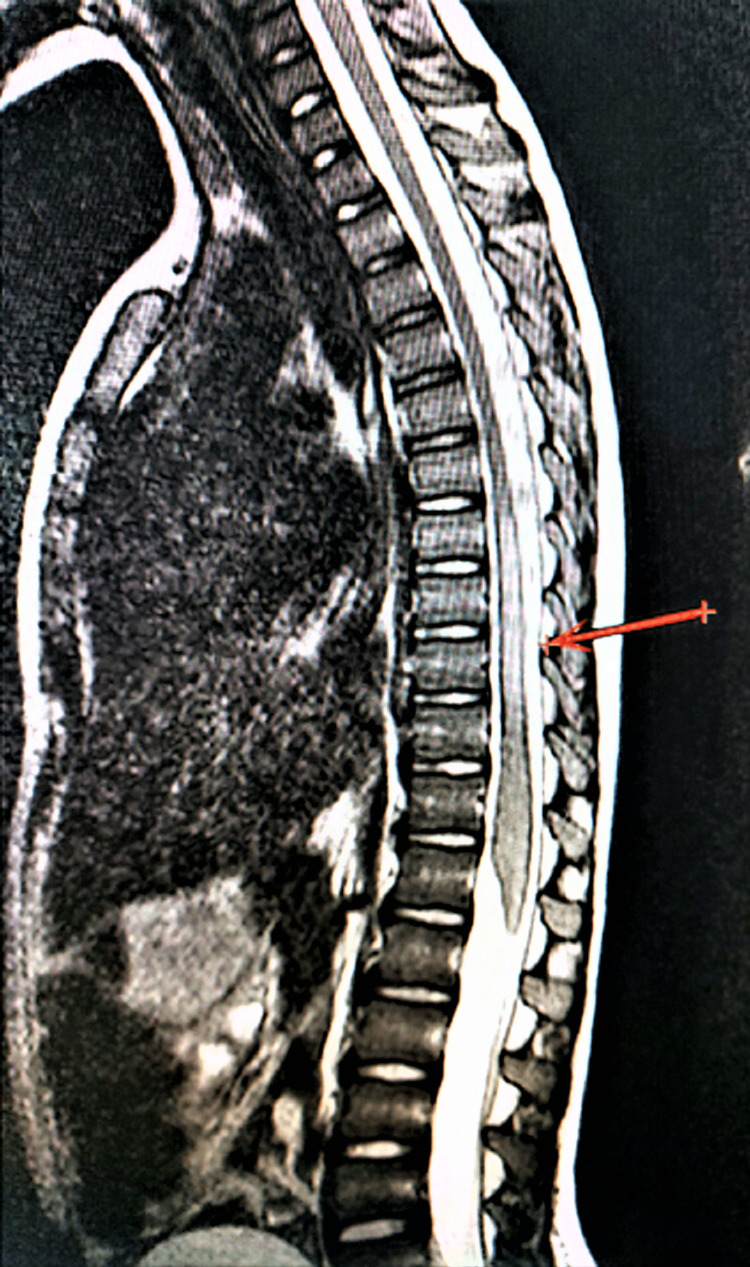
MRI of the spinal cord of a patient at the time of diagnosis of schistosomal myeloradiculopathy T1-weighted sequences after intravenous contrast administration show intense granular and heterogeneous impregnation of the low thoracic spinal cord, conus medullaris, and cauda equina roots in sagittal projections (red arrow).

**Figure 2:**
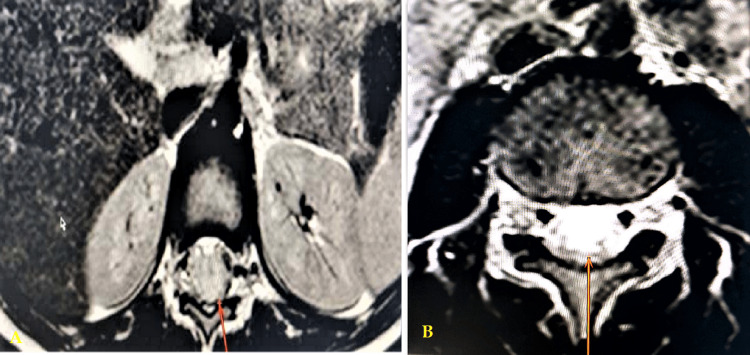
Post-contrast axial MRI T1W images Post-contrast axial MRI T1W images demonstrate the involvement of the ventral surface of the spinal cord A and B (arrows).

MRI findings revealed the presence of lesions in the lower thoracic, lumbar, and/or sacral medullary regions. Additionally, T2-weighted signal hyperintensities were observed in 50% of the cases (Figure 1), Cauda equina lesions were noted in 20% of the cases, and multiple lesions were identified in 30% of the cases (Figure 2).

**Figure 3:**
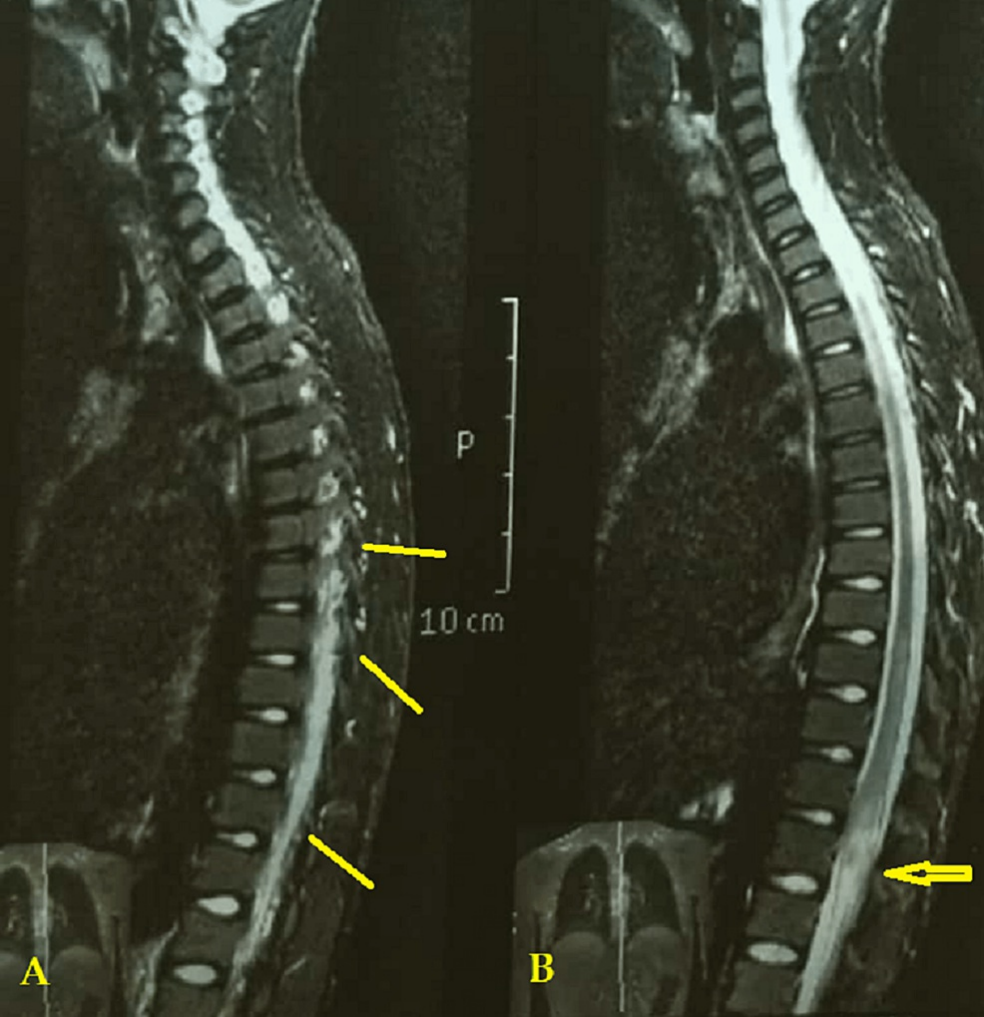
Detailed MRI observations Magnetic resonance imaging showing: A; an impregnation of contrast of the cauda equina with heterogeneous pattern (arrow). B; a dilated conus medullaris (arrow).

Detailed MRI observations included thickening of the spinal roots on T1, a heterogeneous pattern of enhancement with contrasting material, and central linear contrast enhancement surrounded by multiple enhancing punctuate nodules, resembling an "arborized" appearance (Figure 3).

The thoracic (T2) level exhibited the highest level of abnormalities, while the lumbar (L3) level showed the lowest level of abnormalities (Table 2).

**Table 2:** Laboratory and MRI findings of pediatric patients with spinal schistosomiasis Cerebrospinal fluid: CSF, Magnetic Resonance Imaging: MRI.

Variables	N (%)
MRI findings	
A medullary lesion in the low thoracic, lumbar, and/or sacral medulla	5 (50%)
Lesion of the cauda equina	2 (20%)
Multiple lesions	3 (30%)
Urine for Schistosoma exam	
Positive	3 (30%)
Negative	7 (70%)
Stool for Schistosoma exam	
Positive	9 (90%)
Negative	1 (10%)
Spinal cord biopsy	
Yes	2 (20%)
No	8 (80%)
Erythrocyte Sedimentation Rate increase	10 (100%)
CSF findings	
Positive immune assay	8 (80%)
Mild to moderate pleocytosis	8 (80%)
Presence of eosinophils	3 (30%)
Slight to moderate protein increase	7 (70%)
Elevated gamma globulin concentration	7 (70%)
Detected Schistosoma in CSF	
S. mansoni	6 (60%)
S. haematobium	2 (20%)

The diagnosis of spinal NS was confirmed in 80% of cases through CSF serology for schistosomiasis. Additional diagnostic findings included mild to moderate pleocytosis in 80% of cases, CSF eosinophilia in 30%, elevated protein levels in 70%, and elevated gamma globulin levels in 70% of cases. Gram staining and testing for alcohol- and acid-resistant bacilli in CSF found no pathogens. Stool and urine examinations for Schistosoma yielded positive results in 90% and 30% of cases, respectively.

Praziquantel was given orally for three days along with high-dose corticosteroids for all the cases. Follow-up assessments conducted within an average period of 5.6 ± 1.7 months (range: 4-9 months) revealed that one patient (10%) remained paraplegic in the lower extremities, two cases (20%) exhibited partial improvement with residual deficits, including urinary and fecal incontinence, and seven cases (70%) achieved complete resolution of their symptoms (Table 3).

**Table 3:** Treatment outcomes and complications of pediatric patients with spinal schistosomiasis.

Variables	N (%)
Praziquantel	10 (100%)
High dose Corticosteroid	10 (100%)
Need surgical intervention	1 (10%)
Complication	3 (30%)
Type complication	
Paraplegic of lower	1 (10%)
Urinary and bowel dysfunction	2 (20%)
Outcome	
Not improved	1 (10%)
Partial improved	2 (20%)
Fully improved	7 (70%)
Time to the first recovery (day), Mean± SD	16.1±18.9 (range 30– 60)
Time to last follow-up (months), Mean± SD	5.6±1.7 (range 4 - 9)

Comparison between fully improved vs. partially or not improved spinal NS

Statistical analysis showed that a longer duration between starting symptoms to receiving the definitive therapy was longer and statistically significant in partially improved or not improved cases compared to those fully improved (88.3 ± 53.5 days vs. 9.9 ± 2.7 days; p = 0.003) (Table 4).

**Table 4:** Compression between fully improved versus partially improved or no recovery This comparison is according to the time between the start of symptoms to definitive treatment.

Variables	Total	Partially improved or No recovery (N=3)	Fully improved (N=7)	p-value
Mean time from presentation to treatment (days)	33.4±45.6	88.3±53.5	9.9 ±2.7	0.003

## Discussion

In this study, we investigated the patterns and characteristics of pediatric spinal NS in an endemic area of IBB City. The mean age at diagnosis was found to be 10.1±3.2 years. Male patients from rural areas were disproportionately represented, accounting for 90% of the cases. Previous studies have consistently reported a higher prevalence of NS among children and young adults, with a greater proportion of males being affected [9-11]. This observation can be partially attributed to the heightened exposure to risk factors associated with schistosomiasis, such as freshwater habitats hosting snail vectors, within this particular group. Moreover, the geographical distribution of schistosomiasis may contribute to the higher incidence among younger males, as they are more prone to traversing infested areas during periods of flooding when disease transmission is most rampant [5]. Interestingly, a history of exposure to freshwater sources was documented in all of our patients.

The duration between patients' initial presentation and receiving definitive therapy in our study was found to be 33.4±45.6 days (minimum: seven, maximum: 150 days). However, previous studies have demonstrated considerable variability in this timeframe. For instance, a recent study conducted in the UK reported a mean duration of 42.5 days (range: 16-74 days) from presentation to the initiation of definitive therapy [12]. This disparity can be attributed to the significantly lower incidence of schistosomiasis in developed countries. In comparison, a study from Brazil revealed an even wider time range, with a mean duration of 77 days (range: seven days to one year) [13]. Despite the comparatively higher prevalence of schistosomiasis in countries such as Yemen and Brazil, there is a significant delay in reaching a diagnosis, indicating that this condition presents diagnostic challenges, which may arise from overlapping etiologies that manifest similar symptoms. Therefore, it is imperative to consistently gather a comprehensive travel and exposure history, as all the cases included in our study had a documented history of exposure.

Among the cases in our study, the most frequently reported symptoms included bladder dysfunction (100%), intestinal dysfunction (90%), lower limb muscle weakness (80%), fever (80%), rapidly progressive weakness (60%), inability to walk (60%), and abdominal pain (60%). Additionally, paresthesia, deep tendon hyporeflexia, areflexia, and hepatosplenomegaly were observed in 10 (100%), four (40%), two (20%), and two (20%) cases, respectively. These symptoms align with documented literature and are recognized as characteristic manifestations of NS [5,14,15]. It is crucial to consider as a differential diagnosis in patients with a history of exposure to schistosome-infected water, who present with non-localizing neurological symptoms, including sphincter dysfunction, nystagmus, vertigo, hyporeflexia, or sensory impairment [5,16]. Nevertheless, the presentation of NS depends on the involved regions in the nervous system.

The gold standard for diagnosing spinal cord NS is the histopathological examination of clinical specimens obtained through biopsy or necropsy. Pathological findings commonly demonstrate the presence of granulomatous reactions, which can manifest as a mass-like lesion involving the caudal spinal cord, including the conus medullaris and nerve roots of the cauda equina. In more advanced cases, diffuse inflammatory reactions may be observed, accompanied by superimposed necrotic exudative changes or hemorrhage [16]. However, these invasive procedures carry the risk of compromising the neurological function of the patient and should be reserved for cases with uncertainty or lack of response to other diagnostic or therapeutic approaches. The development and availability of non-invasive tests, such as polymerase chain reaction for detecting *S. mansoni* antigens in CSF, would represent a significant advancement in spinal cord NS diagnosis [17,18]. However, conducting such a study was not feasible in our resource-limited setting. In our study, the diagnosis of spinal NS was confirmed in 80% of cases through CSF serology for schistosomiasis. Among these cases, 80% showed increased WBC count, 30% exhibited CSF eosinophilia, and 70% demonstrated elevated proteins and gamma globulin levels. Stool and urine examinations for Schistosoma yielded positive results in 90% and 30% of cases, respectively. In a study by Lambertucci et al., elevated protein levels and lymphocytes count in the CSF were found in 90% of cases, with CSF eosinophilia reported in 40% [17]. Nevertheless, the absence of schistosomal eggs in stool or a negative CSF enzyme-linked immunosorbent assay (ELISA) test does not necessarily rule out the presence of schistosomiasis as they have false-negative results. In such cases, CSF immunological tests utilizing indirect immunofluorescence can be valuable, with a documented positive rate of 70% [19].

MRI assumes a pivotal role in the diagnostic process of spinal NS, wherein it serves to affirm the existence of spinal lesions and bestows invaluable insights into their distinctive characteristics. Abnormal T1-weighted (T1WI) and T2-weighted (T2WI) signals with a heterogeneous pattern of enhancement can be observed on MRI, along with the evaluation of spinal cord compression [13,20]. In our study, MRI findings revealed lesions involving the lower thoracic, lumbar, and/or sacral medulla in 5 (50%), the cauda equina in 2 (20%), and multiple lesions in 3 (30%) of the cases. Generally, previous studies have reported similar patterns of T2WI signal hyperintensities and gadolinium enhancement in cases of spinal NS. However, in our study, we did not observe any fibrotic changes on imaging, which are typically associated with advanced stages of the disease. This observation may potentially indicate that the patients encompassed within our study were evaluated during the earlier stages of spinal NS [21]. The reports regarding the most commonly involved segments in spinal NS are inconsistent across studies. However, a general trend suggests a higher involvement of the lower spinal cord, with multiple segments being affected simultaneously [17]. While computed tomography (CT) scans had demonstrated comparable sensitivity to MRI in detecting spinal atrophy during the later stages of spinal NS, MRI has shown superiority in the earlier stages of the disease [13].

Although spinal cord involvement by schistosomiasis is considered rare, *S. mansoni* is known to cause spinal cord disease predominantly [22]. Infections with *S. mansoni* and *S. haematobium* typically result in myeloradicular damage, while infections with *S. japonicum* primarily lead to cerebral lesions [22]. However, there have been reported cases of spinal NS associated with *S. japonicum* [23]. In our study, *S. mansoni* was the most frequently identified pathogen.

There is currently no definitive consensus on the optimal treatment for spinal schistosomiasis. However, praziquantel remains the most widely used therapy, with a high cure rate of up to 90% in reported cases [24]. In the management of systemic schistosomiasis, single-day administration of praziquantel has been proven to be safe and effective. However, there have been reports of resistance to praziquantel in some African countries. In the treatment of schistosomal myelopathy, a commonly recommended dosing regimen is 40 mg/kg per day for three days for *S. mansoni* and *S. haematobium*, and 60 mg/kg per day for six days for *S. japonicum* [15,20]. However, recent studies suggest that a higher dose of 60 mg/kg per day for three days, administered in two divided doses with a 4-hour interval, may be more effective in treating *S. mansoni* infection in Brazil, achieving a cure rate of 96% [15]. Corticosteroids have also shown promising results in rapidly improving acute schistosomal myelitis, although no controlled studies have been conducted to evaluate their efficacy [15]. Testing for cure is necessary, and a follow-up examination should be performed one month after treatment to assess the efficacy of medical intervention.

During the follow-up period of 5.6±1.7 months in this study, several outcomes were observed. One patient became paraplegic in the lower extremities, while two (20%) cases exhibited partial improvement, experiencing residual deficits including urinary and fecal incontinence. On the other hand, a total of 7 (70.0%) cases showed complete resolution of symptoms. It is noteworthy that the patient who developed paraplegia sought medical attention five months after the onset of symptoms, suggesting a potential delay in response to treatment. Similar findings have been reported in previous studies by Domingues et al. [5] and Badr et al. [8]. Other notable cases described by Kim et al. [25] and Mohamed et al. [26] involved progressive paraparesis resulting from lumbar spinal NS in a 25-year-old and a 4-year-old male, respectively. Most literature reports demonstrate significant improvement when therapy is promptly initiated. Early diagnosis and treatment are crucial for treatment success, as untreated patients face a high risk of mortality or irreversible neurological complications, as observed in our cases [27]. Nonetheless, neurological recovery was observed even in cases when therapy was initiated in the later stages of the disease course [27]. Further studies are necessary to identify potential variables that can help predict the need for prolonged treatment or surgical intervention.

The findings of this study underscore the significance of a comprehensive clinical evaluation, which encompasses a thorough assessment of travel history and potential exposure factors. It is essential for clinicians to consider these aspects as they cannot be substituted by other means. Furthermore, these findings emphasize the inclusion of NS in the differential diagnosis of patients presenting with symptoms indicative of spinal cord involvement in regions endemic to schistosomiasis.

Study limitations

This retrospective study was conducted at a single center, which limits the generalizability of the findings to other settings. Additionally, the sample size was relatively small. Furthermore, the study did not adjust or classify patients based on additional factors such as MRI findings or infection risk factors. These factors can potentially influence the treatment outcome and should be considered in future research. To advance knowledge in this area, future large-scale studies are required to address more specific questions and establish best practices for effective treatment.

## Conclusions

Schistosomiasis should be considered in pediatric patients with myeloradicular manifestations, especially in endemic areas. Early identification can be achieved through a thorough history, prompt imaging, and CSF serology. If schistosomal tests are not immediately available, expert-guided presumptive therapy should be explored to reduce the risk of neurological sequelae.
